# Acceptance, effects, and tolerability in the vaccination process against SARS-CoV-2 among cancer patients in Bosnia and Herzegovina: A single-center cross-sectional study

**DOI:** 10.17305/bjbms.2021.7134

**Published:** 2022-04-08

**Authors:** Timur Cerić, Emir Sokolović, Anes Pašić, Emina Borovac-Gurda, Velda Smajlbegović, Berisa Hasanbegović, Emina Bičakčić Filipović, Elma Kapisazović, Selma Sokolović, Semir Bešlija

**Affiliations:** Clinic of Oncology, Clinical Center University of Sarajevo, Sarajevo, Bosnia and Herzegovina

**Keywords:** COVID-19, vaccination, cancer patients, acceptance

## Abstract

The SARS-CoV-2 pandemic has been the main public health issue since the end of 2019. The vaccination campaign in Bosnia and Herzegovina started in April 2021, with several vaccines available. Our study aimed to evaluate the acceptance, effects, and tolerability of vaccines against SARS-CoV-2 among cancer patients. We conducted a cross-sectional, observational study between 22 October and 30 November 2021, at the Clinic of Oncology, Clinical Center University of Sarajevo. Patients were enrolled during their regular visit to the Clinic of Oncology by agreeing to complete an individual paper questionnaire. The study included 1063 patients with malignant diseases, of whom 681 (64.1%) were adequately vaccinated patients. In the study population, 76.9% of patients reported that they did not experience any side effects due to vaccination, while only 0.5% had side effects, causing a delay in their treatment. Among adequately vaccinated patients, there were 40 patients (3.8%) who were infected with SARS-CoV-2 after the second or booster dose of the vaccine. Five patients (0.5%) were hospitalized due to COVID-19 after being adequately vaccinated. The findings of our study suggest that cancer patients have a higher acceptance of vaccines against SARS-CoV-2 than the general population in Bosnia and Herzegovina. Vaccination side effects are tolerable and do not cause major delays in specific cancer treatment. The protective effects of COVID-19 vaccines in the cancer patients presented in our study are comparable to available results of similar studies, which included the general population.

## INTRODUCTION

The SARS-CoV-2 pandemic has been the main public health issue since the end of 2019. Caused by the acute respiratory syndrome coronavirus-2 (SARS-CoV-2), COVID-19 can present with a diapason of cases – ranging from asymptomatic to severe. The most severe symptoms are respiratory distress, pneumonia, and even death [1].

As of 4 December 2021, 267,865,289 confirmed cases of COVID-19, including 5,285,888 deaths (1.97% of confirmed cases), have been reported worldwide to the World Health Organization (WHO) [2].

The emergence of this pandemic drastically influenced the care of oncology patients worldwide, as well as in Bosnia and Herzegovina [3]. Given that COVID-19 is transmitted mainly by person-to-person contact, public health orders were issued in order to minimize person-to-person interaction [4,5]. The first non-pharmaceutical interventions, colloquially known as lockdowns (stay-at-home orders, quarantines, and police curfews), had been implemented in order to reduce the spread of SARS-CoV-2 starting in China, then across multiple countries [6]. Studies so far have shown that the measures implemented played a significant role in effectively controlling the spread of the COVID-19 pandemic [7].

A single-center study in South Bosnia and Herzegovina, published by Arapović and Skočibušić, showed that the prompt introduction of restrictive socioepidemiological measures resulted in better control of the pandemic in comparison with some higher income countries (e.g., France, Italy, and Spain) during the first several months of the pandemic [8].

A study published by Goletić et al. pointed out the importance of travel-associated disease introduction events. Through the molecular analysis of swab samples from different regions in Bosnia and Herzegovina, they pointed out the possible significance of independent travel modes and their impact on the increased incidence of COVID-19 cases, as well as the importance of these findings in modifying the socioepidemiological measures that are to be implemented [9].

For oncology patients globally, the protective measures meant that diagnostic and surgical procedures were delayed, treatment plans were altered in order to minimize visits to the clinics, and routine follow-ups were postponed [8]. Many patients had to receive treatment in- clinic, in larger hospitals, or clinical centers meaning greater exposure and higher risk for getting infected with SARS-CoV-2. Changes in the treatment regimen of these patients may have led to disease progression or even worse outcomes [9, 10]. Every oncology center made a management strategy for the spread of COVID-19 in order to reduce the delay of treatment. The COVID-19 pandemic has shown a significant impact on cancer patient care worldwide. The ONCOCARE-COV study, published by Brugel et al., shows the dramatic impact the COVID-19 pandemic had on different levels of oncology patient care. A relative decrease in chemotherapy and radiotherapy treatment was observed, as well as a significant negative impact on screening, cancer diagnostics, and surgical treatment of oncology patients [11]. Similar results can be found in other European countries such as Belgium, the UK, Spain, as well as in the US [12-17]. Because majority of systems were faced with limited healthcare personnel during the pandemic, an international collaborative group recommended a prioritization plan to maximize health benefits, considering the patient, their disease, and its prognosis [11-18]. Between March and May 2020, 77 of the 8657 patients scheduled for therapy at the Clinic of Oncology Sarajevo had their treatment delayed due to a positive anamnesis or a high body temperature. Out of the 40 symptomatic patients tested, infection with SARS-CoV-2 was confirmed in 2 [11]. Šušak et al. published a single-center study in Konjic, Bosnia and Herzegovina, demonstrating the correlation between symptoms and IgG seroconversion to SARS-CoV-2 one year after infection, with patients having positive IgG serology one year after contact. Symptoms of high fever and headache could be possible indicators of a better immune response as they have shown correlation with IgG levels. The study has also shown a significant increase in antibody titers of vaccinated participants one year after infection which could possibly point to better protection against reinfection [19].

Oncology patients on active treatment were quickly considered as a potentially vulnerable population, especially individuals with risk factors such as therapy-related immunosuppression, comorbidities, and age [12,20]. Conducted studies clearly showed that patients that go in for inpatient or outpatient treatment have an increased risk of COVID-19 infection, concern about the risk of infection with COVID-19 when coming to the hospital, and some patients even showed reluctance to proceed with treatment [13,21]. Thus, leading oncology societies recommend that cancer patients on active treatment, those starting treatment, and those that have been treated in the past six months be prioritized for vaccination [22].

Protective behavior is crucial to managing a pandemic, and effective immunization could bear the most promise for resolving the health issue of COVID-19 pandemic [14,23]. During 2020, several vaccines were being developed in multiple countries, and by the end of the year, results of the phase 3 trial had been published which resulted in the approval of the vaccines against COVID-19 [15-17, 19, 21].

Vaccination against COVID-19 commenced worldwide at the beginning of 2021. The Strategic Advisory Group of Experts on Immunization issued a framework for the prioritization of COVID-19 vaccination considering cancer patients a high priority population, a plan that was also implemented in Bosnia and Herzegovina [22,24-26]. Vaccination campaign in Bosnia and Herzegovina started in April 2021 with several vaccines available: Sinopharm (BBIBO), CoronaVac (Sinovac), ChAdOx1 nCoV-19 (Oxford/AstraZeneca), and later on, BNT162b2 (Pfizer/BioNTech) [27-30]. It must be noted that a significant number of individuals were vaccinated outside of the country before the vaccination campaign had started in Bosnia and Herzegovina.

Up until the 9 December 2021, a total of 8,158,815,265 vaccine doses have been administered, as reported to the WHO [31]. Data for Bosnia and Herzegovina showed a total of 280,469 confirmed cases of COVID-19 reported, out of which 12,882 of these cases resulted in death. Up until the 4 November 2021, a total of 1,553,874 vaccine doses have been administered. In total, 833,233 individuals have been vaccinated with at least one dose, and 720,631 individuals have been fully vaccinated [31,32].

According to the Institute for Public Health of the Federation of Bosnia and Herzegovina, in the period from 3 March to 5 December 2021, a total of 995,646 persons were vaccinated, out of which 525,844 received at least one dose, 461,597 two doses, and 8205 persons received the third dose [32].

According to the current data, several studies have been conducted to assess the cancer population’s attitude toward COVID-19 immunization. Several cross-sectional surveys have been conducted in order to better understand the acceptance of the COVID-19 vaccination of patients with malignant diseases. Most studies have shown that the majority of patients are willing to get vaccinated [33-36].

The aim of our cross-sectional, observational study was to evaluate the acceptance, effects, and tolerability of vaccines against the SARS-CoV-2 among cancer patients.

## MATERIALS AND METHODS

This was a cross-sectional, observational study, conducted between 22 October and 30 November 2021, at the Clinic of Oncology, Clinical Center University of Sarajevo. The study included 1063 patients with malignant disease that have been visiting inpatient or outpatient departments of our clinic for treatment, follow-up, and consultations. They were enrolled during their regular visit to the Clinic of Oncology by agreeing to complete an individual paper questionnaire on personal demographic information (initials, age, gender, and area of living), information on previous COVID-19 infection, COVID-19 vaccination acceptance, vaccination status, side effects of COVID-19 vaccination, and eventual delay of oncologic treatment caused by vaccines side effects. During the scheduled appointment, the attending physician completed the questionnaire with information about the diagnosis, presence of metastatic disease, and modality of the therapy. Vaccinated patients in our study received one of the following vaccines against SARS-CoV-2: BNT162b2, ChAdOx1 nCoV-19, Sinopharm, or CoronaVac.

### Ethical statement

Ethical approval was obtained from the Hospital Ethics Committee at the Clinical Center University of Sarajevo (number 1178/21). Participation in the study was voluntary. Participants were assured of anonymity and confidentiality of their responses. Refusals were not documented. Patients received no financial compensation. The questionnaire was approved by the Institutional Ethics Committee.

### Statistical analysis

We defined the descriptive measures, including absolute value and percentages. The chi-square test of independence was used to evaluate the relationship of categorical variables between adequately and inadequately vaccinated patients. The chi-square test of independence was used to evaluate the presence of side effects and type of administered vaccine. Post hoc chi-square testing was performed to analyze adjusted residuals and to identify cells with statistically significant z-scores in cross tabulation. *p* < 0.05 was an indicator of significance. IBM SPSS Statistics v. 23.0 was used for the statistical analysis.

## RESULTS

Our study included 1063 patients who were treated or examined at the Clinic of Oncology, Clinical Centre University of Sarajevo during October and November 2021.

The mean age of all patients included in our study was 61.9 (SD = 11.5) years and study population consisted of 65.7% of female patients and 34.3% of male patients.

The characteristics of the study population are presented in Table 1.

**TABLE 1:**
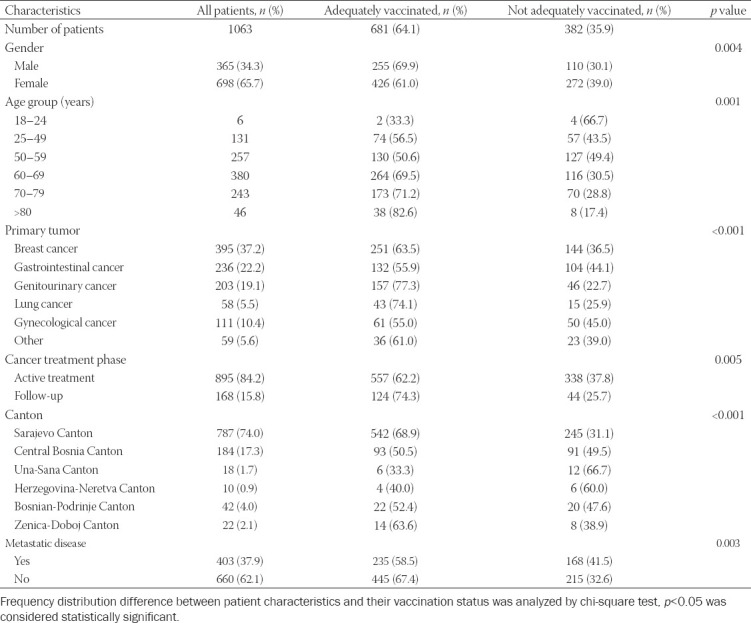
Characteristics of patients included in the study

In our study population, there were 339 (31.9%) unvaccinated patients and 43 (4.0%) patients who received the first dose of a vaccine. These patients were classified as inadequately vaccinated patients in further analysis. Patients who were classified as adequately vaccinated were those who received a second dose of a COVID-19 vaccine (63.9% of patients) or a booster (third) dose (0.2% of patients). The frequency distribution difference of vaccinated patients across cantons (areas of living) was statistically significant (*p* < 0.001). The highest percentage of vaccinated patients was in the Sarajevo Canton (68.9%), while the lowest percentage was in the Una-Sana Canton (33.3%).

The most commonly administered vaccine was BNT162b2 – 53% of the patients, while 23.3% of patients were vaccinated with ChAdOx1 nCoV-19 vaccine, 20.7% with the Sinopharm vaccine, and 3% chose the CoronaVac vaccine.

SARS-CoV-2 infection prior to completing the questionnaire was reported by 261 (26.5%) patients.

**FIGURE 1:**
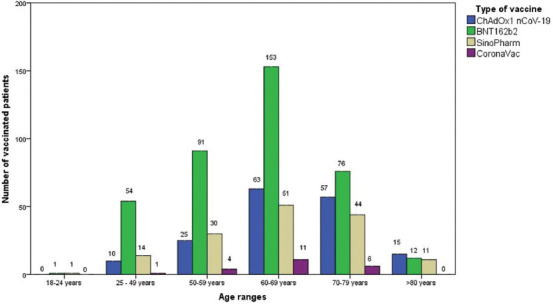
Frequency of different types of administered vaccines in different age groups of patients.

BNT162b2 was the most commonly administered vaccine in almost all age groups, except in patients over 80 years where the most common vaccine was ChAdOx1 nCoV-19 (Figure 1).

In our study population, 76.9% of patients reported that they did not experience any side effects of vaccination, while only 0.5% of patients had a delay in their treatment due to side effects. The most commonly reported side effect was local pain at the site of vaccine injection, and it was reported in 12.3% of patients. Besides local pain, fever was reported in 5.5% of patients, myalgia in 5.0% of patients, fatigue in 4.1% of patients, bone pain in 2.9% of patients, and 1% of patients complained about having nausea or vomiting after vaccination. Table 2 presents the most common side effects reported with respect to the vaccine administered. Using post hoc chi-square testing and values of adjusted residuals (z-scores) in cross-tabulation, we have identified that side effects of vaccination were most commonly reported in patients vaccinated with ChAdOx1 nCoV-19 vaccine (z-score =2.1; *p* = 0.03) and that patients without side effects of vaccination were most commonly vaccinated with Sinopharm vaccine (z-score = 3.8; *p* < 0.001). Local pain was the most commonly reported side effect. About 12.3% of patients reported local pain and it was most commonly present in patients vaccinated with the BNT162b2 vaccine (z-score = 3.8; *p* < 0.001). ChAdOx1 nCoV-19 vaccine was the most commonly administered vaccine in patients who reported fever as a side effect (z-score = 3.8; *p* < 0.001).

**TABLE 2:**
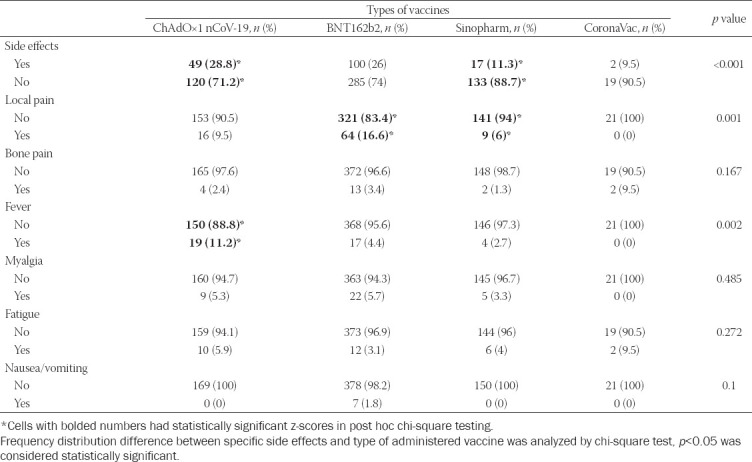
Side effects of specific types of vaccines administered to patients

Besides side effects reported in Table 2, three patients reported headache as side effect of vaccination. Two of them were vaccinated with the BNT162b2 vaccine and one with the ChAdOx1 nCoV-19 vaccine. No serious adverse events of COVID-19 vaccines were reported by the patients.

Among adequately vaccinated patients, there were 40 patients (3.8%) who were infected with SARS-CoV-2 after the second or booster dose of the vaccine. The mean number of months of SARS-COV-2 infection after being adequately vaccinated was 3.35 (SD = 1.77). Five patients (0.5%) were hospitalized because of COVID-19 after being adequately vaccinated.

The type of vaccine administered was not significantly related with patients who were infected with SARS-CoV-2 or hospitalized due to COVID-19 after full vaccination.

The presence of metastatic disease did not have a statistically significant relation with being infected with SARS-CoV-2 or hospitalized due to COVID-19 after adequate vaccination.

## DISCUSSION

The first cases of COVID-19 in Bosnia and Herzegovina were reported at the beginning of March 2020. The vaccine campaign in Bosnia and Herzegovina had started later than in other European countries, probably due to the late arrival of the vaccines to our country [32,37-39]. In order to minimize the risk of COVID-19 infection and severe complications in this vulnerable population, cancer patients were prioritized as a group and encouraged to get vaccinated by the ongoing campaign for vaccination through media outlets along with recommendations from their oncologists. Variations in the vaccination rate of the population across different geographical areas could be attributed to the different quality and intensity with which local experts implemented vaccination campaigns against the SARS-CoV-2.

Several studies reporting on the safety and efficacy of COVID-19 vaccines among the cancer patient population have been published. Yasin et al. published a multicenter cohort study showing that cancer patients have significantly lower seropositivity rates compared to non-cancer patients (85.2% and 97.5%, respectively) when vaccinated with the CoronaVac vaccine. These findings were not surprising, considering that cancer patients are immunosuppressed, which, therefore, have a negative effect on the immune response. The study also confirmed the safety and efficacy of applying the CoronaVac in cancer patients [40]. Jackson et al. found a seropositivity rate of 86.9% in cancer patients vaccinated with Sinopharm inactivated vaccine, also finding that low seropositivity rates are mostly found in elderly cancer patients, those on active treatment, and patients with hematologic malignant disease [41]. Massarweh et al. found that 90% of patients receiving systemic anticancer treatment had an adequate immune response, but also significantly lower antibody titers compared to a healthy control group after receiving the BNT162b2 vaccine. The lowest antibody titers observed were in patients receiving chemotherapy in combination with immunotherapy [42].

According to the WHO, 720,631 individuals were fully vaccinated in Bosnia and Herzegovina up to November 2021, while 882,641 individuals received at least one dose. [43].

According to the data from the Institute for Public Health of the Federation of Bosnia and Herzegovina, in the period from 3 March 2021 to 16 January 2022, only 28.20% of the population has been fully vaccinated, while 7.47% received a booster dose [32,39]. Marijanovic et al. showed that most cancer patients (62.2%) had a hesitancy toward immunization with COVID-19 vaccines through their cross-sectional study conducted at the Clinic of Oncology, Mostar, Bosnia and Herzegovina [44]. Although we had a small sample of patients from this area of Bosnia and Herzegovina, our results are compatible since 40% of patients from the Herzegovina-Neretva Canton were adequately vaccinated. However, we should mention that this study was conducted during February 2021, when data on the safety of vaccination in cancer patients were limited.

A French cross-sectional study has shown that 53.7% of oncology patients, on active treatment or active surveillance, were likely to be vaccinated, while 29.7% considered themselves not ready yet [45].

Similar results have been documented in a Portuguese study by de Sousa et al. where a majority of cancer patients (84%) on immunosuppressive therapy had the intention to be vaccinated, as well as a Lebanese study where 55% of patients had shown willingness for COVID-19 vaccination [46].

A cross-sectional study carried out in Serbia has shown that 41.72% of cancer patients were vaccinated, while 17.67% wanted to be vaccinated as soon as possible. More than half of the patients not wanting vaccination stated that they wish to be vaccinated after their cancer treatment, which may point to a fear of delaying active cancer treatment due to possible side effects [35].

However, data from our study show that 64.1% of patients were adequately vaccinated and these results outweigh the first data on vaccination hesitancy among cancer patients. In the majority of studies conducted, the main reasons for patients’ unwillingness to get vaccinated are uncertainty about possible side effects or concern that vaccines may impair cancer treatment efficacy and outcomes. This suggests that these may be the reasons why patients on follow-up were significantly more vaccinated than patients on active treatment in our study (74.3% and 62.2%, respectively) [47].

When divided by the area of living, the highest percentage of vaccinated patients was in the Sarajevo Canton (68.9%). According to the Institute for Public Health of Canton Sarajevo, 50.1% of the Sarajevo Canton population has been fully vaccinated with a COVID-19 vaccine between March 2021 and January 2022 [48]. Comparing these two results, we can conclude that the encouragement and recommendation of oncologists at local clinics for vaccination against SARS-CoV-2 were successful since the percentage of vaccinated cancer patients from Canton Sarajevo is higher than in the general population. The lowest percentage of vaccinated patients was in the Una-Sana Canton (33.3%). The difference in the percentage of vaccinated patients could be caused by the fact that the Sarajevo Canton is located in a more urban setting, and thus having the population greater exposure to the vaccination campaigns conducted, along with vaccine promotion through health institutions and various forms of media.

As with most studies published to this day, data show that there is a significant correlation between age and vaccine acceptance [49]. We note that the majority of patients vaccinated were 50 years old and above, while only 33.3% of patients aged 18-24 years were adequately vaccinated which may imply that the older part of the population consider themselves to have a greater benefit from vaccination, lowering so their risk of COVID-19 complications.

In our study, 53% of patients received BNT162b2, while in the neighboring Serbia was reported that the majority of cancer patients received the Sinopharm vaccine [28-30,35].

Since cancer patients were not included in vaccine clinical trials, limited data about vaccine tolerability for this part of population are available [26,50].

Meta-analysis on the safety of COVID-19 vaccines in cancer patients suggested that vaccination appeared to be generally very safe, with mostly mild and moderate adverse effects reported. None of the included studies have described serious adverse events [51].

In our study population, 76.9% of patients reported that they did not have any side effects of vaccination, while only 0.5% of patients had a delay in their treatment caused by the side effects.

The most common side effect was local pain at the injection site, reported in 12.3% of patients, out of whom the majority received the BNT162b2 vaccine.

Our study results correspond to the other results where the incidence of side effects in patients receiving the Sinopharm vaccine was lowest compared to other types of vaccines [35]. Reports of fever were the highest amongst patients receiving the ChAdOx1 nCOV-19 vaccine (11.2%), which was significantly higher compared to other vaccines.

Tenforde et al. presented that vaccine effectiveness was significantly reduced for patients with immunocompromising conditions (59.2%) compared to individuals without an immunocompromising condition (91.3%). They showed that, when restricted to immunocompromised patients with an active solid organ, or hematologic malignancy, or solid organ transplant, vaccine effectiveness was 51.2%. In this study, authors also reported that 20% of patients who developed COVID-19 symptoms after being vaccinated were patients with active solid organ or hematologic malignancy. In the group of patients who developed COVID-19 after being adequately vaccinated, the median time between the final vaccine dose and symptom onset was 44 days [52]. In our study, only 3.8% of patients were infected with SARS-CoV-2 after the second or booster dose of the vaccine and the mean number of months of SARS-CoV-2 infection after being adequately vaccinated was 3.35 months (SD = 1.77).

According to the report of the Institute for Public Health of Canton Sarajevo, 76% of hospitalized patients are unvaccinated, while only 24% of hospitalized patients have been fully vaccinated [48]. The Israeli study shows that only 7.7% of patients hospitalized with COVID-19 were fully vaccinated with 7 or more days after the second dose of vaccine. This pointed to vaccine effectiveness in comparison to 71.8% of COVID-19-related hospitalizations in which patients were not vaccinated [53]. In our study population, five patients (0.5%) were hospitalized because of COVID-19 after being adequately vaccinated and they represent 12.5% of patients who developed COVID-19 after being adequately vaccinated.

The limitation of our study was that it was conducted in a single center, although oncology patients from different geographical areas of Bosnia and Herzegovina are getting treated at our center. Furthermore, the period between the initiation of the vaccine campaign in Bosnia and Herzegovina and initiation of our study was too short to fully assess the effects of vaccines regarding developing COVID-19 and frequency of hospitalizations caused by COVID-19 among vaccinated patients. Another limitation of our study was the method of patient enrolment in the study, since it was based on the voluntary completion the questionnaire during their regular visit at our clinic. We can assume that a certain number of patients with poor COVID-19 outcome did not show up for regular check-ups and therefore were not a part of the analyzed population within our study.

## CONCLUSION

The findings of our study suggest that cancer patients have a higher acceptance of vaccines against SARS-CoV-2 than the general population in Bosnia and Herzegovina. Vaccination side effects are tolerable and do not cause any major delay in specific cancer treatment. The protective effects of COVID-19 vaccines in the cancer patients presented in our study are comparable to available results of similar studies which included the general population.

In order to have more reliable conclusions about the effectiveness and safety of the use of COVID-19 vaccines among cancer patients, it is necessary to conduct a number of studies of different designs that will have longer follow-up periods. However, at this point, we have enough available evidence to convincingly recommend COVID-19 vaccination to cancer patients.

## SUPPLEMENTAL DATA

Questionnaire S1

PLEASE ANSWER THE FOLLOWING QUESTIONS:

1. Patient initials_____________________________

2. Age of patient (years)_____________________________

3. Gender

a) M
b) F

4. Are you currently receiving oncology therapy?

a) Yes
b) No

5. Have you had COVID-19 before vaccination?

a) Yes
b) No

6. Vaccination status

a) Vaccinated – III doses
b) Vaccinated – II doses
c) Vaccinated – I dose
d) Unvaccinated

7. Date of the administration of the first dose of vaccine?___________________________

8. Type of vaccine:

a) BNT162b2 (Pfizer/BioNtech)
b) ChAdOx1 nCoV-19 (Oxford/AstraZeneca)
c) Sinopharm (BBIBO)
d) CoronaVac (Sinovac)

9. Side effects of vaccine:

a) Without any side effects
b) Local pain
c) Fever
d) Muscle pain
e) Bone pain
f ) Fatigue
g) Nausea and vomiting
h) Other symptoms (please describe)___________________________________

10. Have you had COVID-19 after vaccination?

i) Yes
j) No
k) (If answer is YES please specify exact date of positive PCR SARS-CoV-2 test __________________)

11. Have you been in the hospital due to COVID-19 infection after vaccination against SARS-CoV-2?

l) Yes
m) No

12. Whether your oncology therapy has been delayed due to the side effects of the vaccine against SARS-CoV-2?

n) Yes
o) No

13. Canton of living?

a) Sarajevo
b) SBK
c) USK
d) HNK
e) BPK
f ) ZDK
f ) ZDK
g) ZHK

TO BE COMPLETED BY A DOCTOR WHO PERFORMS AN EXAMINATION

1. Patient diagnosis________

2. Metastatic disease?

a) Yes
b) No

3. Setting of therapy

a) Adjuvant
b) Neoadjuvant
c) Metastatic milieu

4. Modality of therapy

a) Chemotherapy
b) Target therapy
c) Hormonal therapy
d) Immunotherapy
e) Radiotherapy
